# Nonalcoholic fatty liver disease in children with obesity– observations from one clinical centre in the Western Pomerania region

**DOI:** 10.3389/fendo.2022.992264

**Published:** 2022-10-31

**Authors:** Katarzyna Marcinkiewicz, Anita Horodnicka-Józwa, Tomasz Jackowski, Kamila Strączek, Agnieszka Biczysko-Mokosa, Mieczysław Walczak, Elżbieta Petriczko

**Affiliations:** Department of Pediatrics, Endocrinology, Diabetology, Metabolic Diseases and Cardiology of the Developmental Age, Pomeranian Medical University, Szczecin, Poland

**Keywords:** NAFLD, fatty liver, obesity, ALT, glucose intolerance

## Abstract

Nonalcoholic fatty liver disease (NAFLD) is a growing health problem in the pediatric population, due to the constantly increasing percentage of children with obesity. The objective of the study was to assess the occurrence of NAFLD based on ultrasound (USG) analysis and the use of alanine aminotransferase (ALT) in children with overweight and obesity depending on glucose tolerance. Medical records of 228 consecutive patients aged 2-18 years with overweight and obesity were reviewed retrospectively. Based on the oral glucose tolerance test children were divided into groups according to the severity of carbohydrate metabolism disorders. ALT, lipid parameters and insulin sensitivity indices HOMA, Matsuda and Quicki were analyzed. NAFLD was diagnosed (based on the USG) in 51 patients (23.61%) - the incidence in the impaired glucose tolerance (IGT) and type 2 diabetes (T2DM) group was significantly higher when compared to ones without glucose intolerance. Because of extreme values of metabolic parameters in TDM2 children being outliers, they were not considered in the statistical analysis of the study. 22 (11.58%) patients had elevated ALT values, of which 12 (54.55%) had hepatic steatosis features on ultrasound. 72.73% (n=32) patients with fatty liver features on USG had ALT values considered normal with cut-off point 42 U/l accepted in this study. Almost every fourth obese child in the study group presents features of fatty liver in ultrasound examination. Although ultrasound is not recommended by North American *Society* For Pediatric Gastroenterology, Hepatology &Nutrition(NASPGHAN) for the diagnosis of NAFLD in children, it allows identifying a high percentage of children with features of fatty liver. This percentage increases significantly in children with glucose intolerance.

## Introduction

The increasing number of overweight and children with obesity causes nonalcoholic fatty liver disease (NAFLD) to be one of the most common pathologies of this organ (1). Its occurrence can lead to hepatic fibrosis and cirrhosis (2). In the last decades NAFLD has been the leading cause for liver transplants in adult patients. Due to the severity of the issue, in 2017 NASPGHAN has published recommendations regarding the diagnosis and treatment of nonalcoholic fatty liver disease in children (3).

Pathogenesis of nonalcoholic fatty liver disease’s development is complex (4). The emphasis is put both on genetic, as well as metabolic factors (insulin resistance, hyperglycemia, dyslipidemia, cytokine overproduction, oxidative stress). Accumulation of triglycerides in the liver is caused by three factors: lipolysis in the adipose tissue, *de novo* synthesis caused by excess carbohydrate consumption in the diet and dietary lipids. In the setting of overnutrition and obesity, hepatic fatty acid (FA) metabolism is altered, commonly leading to the accumulation of triglycerides within hepatocytes, and to a clinical condition known as NAFLD (5). It has been proven, that the association between dietary fructose and NAFLD is stronger than with glucose. In the recent years the consumption of fructose has significantly increased in the form of high-fructose corn syrup (HFCS) and mixtures of fructose and glucose monosaccharides (6, 7). Incorrect eating habits and excess carbohydrate consumption lead to insulin resistance, which is proven to be one of the important risk factors for NAFLD (2, 8). Bugianesi et al. suggest, that an increased flow of free fatty acids to the liver caused by peripheral insulin resistance causes its steatosis (9). In the adipose tissue of patients with diabetes, disruptions in translocation and activation of glucose transporters have been found. Their lower number and incorrect function have also been found in obese patients. The European Association for the Study of the Liver-The European Association for the Study of Diabetes and The European Association for the Study of Obesity (EASL-EASD-EASO) recommendations include active seeking for impaired glucose tolerance in patients with NAFLD, as well as for NAFLD in type 2 diabetes patients (10).

Alanine and aspartate aminotranferases (AST) are markers of hepatic cell damage, ALT more so than AST. ALT isawidely used marker of hepatic steatosis. The upper limit for children in different age groups allowing to assess the risk of NAFLD is debatable (11, 12). Sensitivity of ALT alone is lower than when using imaging studies and histopathology. NASPHGAN recommendations suggest alanine aminotransferase assessment as a screening test. The laboratory norm has been established using ALT levels in healthy children in accordance to age, and set as 22 U/l for girls and 26 U/l for boys (3). According to those guidelines, norms set by local laboratories should not be taken into account. ALT levels exceeding the norm twofold for longer than 3 months should encourage further investigation, where NAFLD and other chronic inflammatory liver diseases. Studies evaluating the usefulness of aspartate transaminase and γ-glutamyltransferase (GGTP) as a diagnostic marker of NAFLD in children have not yet been performed. However, some authors emphasize, that elevated levels of ALT, AST and GGTP are associated with worse histopathology of the liver biopsies. Elevated AST and GGTP levels with normal ALT may suggest liver diseases other than NAFLD (13). Ultrasonography alone, without checking markers in the serum, is not considered a screening test.

NASPHAN recommendations authors emphasize, that the screening test, i.e. ALT level assessment, should be performed from the age of 9-11 in children with obesity with body mass index(BMI) ≥ 85^th^ percentile and additional risk factors, such as central obesity, insulin resistance, prediabetes and diabetes, dyslipidemia, sleep apnea or family history of NAFLD. Due to the fact, that an increasing fraction of the pediatric population is at risk of being overweight or obese because of changes in eating habits and insufficient physical activity, early diagnosis of NAFLD is necessary. The search for the best markers and risk factors for the disease lies in the interests of many investigators. In the recent years an increasing number of publications concerning this problem has appeared.

Our hypothesis is that simple, non-invasive methods of routine investigation may be useful and sufficient to identify severe metabolic complications in children with obesity. The aim of the study is to assess the incidence of hepatic steatosis by the way of routine investigations in overweight children, including abdominal ultrasound (USG) and alanine aminotransferase (ALT) levels, in correlation to different grades of carbohydrate metabolism disorders.

## Patients and methods

Medical records of 228 consecutive patients in the age of 2-18 years with overweight and obesity were retrospectively analyzed. The study was approved by the Pomeranian Medical University Bioethical Committee on April 14 2004, decision number BN-001/67/04. None of the children were previously diagnosed because of overweight. Neither glucose metabolism disorders nor clinical signs of diabetes have been observed in the children before. All those children were hospitalised in one day clinic of the referential Pediatric Endocrinology Clinic for the Western Pomerania region, inhabited by almost 2 million people, over the course of four years.

Obesity and overweight were defined with BMI SDS. To establish norms for Polish population for body weight, height ant BMI percentile charts by Palczewska and Niedzíwiecka were used (14). Children with secondary causes of obesity (genetic, endocrine disorders e.g. hypothyroidism, Cushing’s disease etc., iatrogenic obesity) as well as children with active hepatotropic virus infections, autoimmune liver diseases and congenital metabolic defects were excluded from the study. Children with secondary causes of obesity (genetic, endocrine disorders e.g. hypothyroidism, Cushing’s disease etc., iatrogenic obesity) were excluded from the study. The primary aim of the study was to assess the risk of obesity-related metabolic complications. Anthropometric parameters were measured (body height, body weight, BMI) as well as puberty stage according to Tanner’s scale. Based on the results of oral glucose tolerance test (OGTT) the study group was divided in four groups: with no carbohydrate metabolism disorders, with impaired fasting glucose (IFG), with impaired glucose tolerance (IGT) and with type 2 diabetes (T2DM). In OGTT both glucose and insulin levels were analyzed in 0 and 120 minute of the test. In the whole study group an abdominal ultrasound with hepatic steatosis assessment was performed, as well as laboratory studies consisting of hepatic enzymes levels, i.e. alanine and aspartate aminotransferase (ALT and AST), lipid profile, i.e. cholesterol level, high-density lipoprotein (HDL), low-density lipoprotein (LDL) and triglycerides (TG). Insulin resistance indices have been calculated: homeostasis model assessment of insulin resistance (HOMA-IR) (15, 16), Matsuda index, quantitative insulin sensitivity check index (Quicki). To assess the hepatocyte function ALT and AST serum levels were measured, using kinetic method. The norm has been set as below 39 U/l for AST and below 42 U/l for ALT. Total cholesterol levels were assessed using the enzymatic-colorimetric method with cholesterol esterase. TG levels were assessed using the enzymatic-colorimetric method with phosphoglycerol esterase. HDL levels were measured using direct method with enzymes modified with poliethylene glycol. LDL levels were measured using direct method with catalase. All lipid profile analyses were performed on Roche Cobas C501 device. Total cholesterol levels below 170 mg/dl were accepted as normal, 170-199 mg/dl as high normal and above 199 mg/dl as high. HDL cholesterol levels higher than 35 mg/dl have been considered as normal, while the values equal and below 35 mg/dl as low. LDL levels below 110 mg/dl were considered as normal, levels of 110-129 mg/dl as high normal and above 129 mg/dl as high. Reference values for triglyceride levels were different for girls and boys. For girls values lower than 130 mg/dl were considered normal, whereas in boys it was values below 120 mg/dl. Values 130-150 mg/dl and 120-150 mg/dl were considered high normal for girls and boys respectively, while values above 150 mg/dl were considered high for both sexes.

To measure insulin resistance indirect methods were used. Based on glucose and insulin serum levels measured during OGTT insulin resistance and insulin sensitivity indices were calculated:

Quantitative Insulin Sensitivity Check Index (Quicki) = 1/log fasting insulin (μIU/mL) + log fasting glucose (mg/dl).Values below < 0.34 suggest insulin resistance.

Homeostasis Model Assessment of Insulin Resistance (HOMA-IR) = fasting glucose (mg/dl) x fasting insulin (μIU/mL)/405. It is assumed, that values >2.5 for I and V stage of the puberty according to Tanner score suggest insulin resistance, and for II-IV stage of the puberty values >3.5 suggest insulin resistance.

Matsuda Index = 100000/√fasting insulin (μIU/mL) x fasting glucose (mg/dl) x mean OGTT glucose (mg/dl) x mean OGTT insulin (μIU/mL).Values<7.3 suggest insulin resistance.

Abdominal ultrasound was performed in all subjects. The examinations were performed on Philips Pure Wave CX50 using convex C5-1 for older children and C8-5 for younger children. All examinations were performed on the same device by two experienced ultrasonographists. Hepatic steatosis was graded using US-FLI semi-quantitative method (17), however the subjects were not divided according to the grade of steatosis, only the presence or absence thereof was considered.

All children with obesity hospitalised in the Clinic took part in dietary schooling and had access to psychologic support. Children’s eating habits were analyzed using authors’ proprietary nutrition questionnaire. Eating habits were rated based on 24-hour eating history, repeated 3 times (3 days of the week chosen by the child’s mother, including 1 day off school). To assess the portion sizes, “Album of photographs of food and dishes” issued by National Food and Nutrition Institute (NFNI) was used (18). Children below the age of 13 filled the questionnaire with their mothers, while children above 13 did it alone or with parental help. To calculate the caloric intake and basic macronutrients of the children’s diet, a computer program DIETA.5 created by NFNI was used. Caloric demand was calculated for every patient individually in accordance to NFNI nutrition norms, considering ideal body weight, age, sex and physical activity level (19)]. Suggested macronutrient ratio of total daily caloric intake, i.e. proteins (12.5%), digestible carbohydrates (57.5%), simple carbohydrates (15%), overall fats (30%) and saturated fats (10%) was used to create dietary recommendations for subjects in this study. Because of extreme values of metabolic parameters in TDM2 children being outliers, they were not considered in the statistical analysis of the study.

### Methods of statistical analysis

Based on the gathered results a database was created in Microsoft Excel. Further statistical analysis was performed in the Stata 11.0 program. The Kolmogorov-Smirnov test was used to verify the normality of distributions of continuous variables. Those variables were then described as mean values and standard deviations. Statistical differences between two groups were then checked with t-Student test and Mann-Whitney test. Non-continuous variables were described as quantity and frequency. The analysis of relationships between non-continuous variables was carried out using Pearson’s χ^2^ test or Fisher’s test. To analyze the relationships between continuous variables Pearson’s correlation was used. The results were described as probability – p, correlation coefficient – r, and regression lines. To estimate the risk of a pathology depending on its risk factors a model of logit regression was used. The results were described by calculating the odds ratio (OR) along with 95% confidence interval. Probability was calculated in this model using Pearson’s χ^2^ test or two-sided Fisher’s exact test. If OR>1, the analyzed factor increases the risk. If OR<1, the analyzed factor decreases the risk. For the whole model R values (multidimensional correlation coefficient) and p (probability) were also calculated. For all of the performed analyses, statistical significance was considered at p values ≤ 0.05.

## Results

The study group consisted of 228 children hospitalised because of overweight and obesity. Within this group there were 118 girls (51.75%) and 110 boys (48.25%). The average age was 10.4±3.6 years.122 of the children were prepubertal (53.51%).

The average body weight of the examined children was 62.7 ± 23.9 kg, while the average body weight SDS was 2.98 ± 0.91. The average BMI body mass index value was 27.83 ± 4.7 kg/m^2^, the average BMI SDS 2.98 ± 0.74. In 83.77% (n = 191) subjects there were no changes in glucose tolerance, 3.07% (n = 7) subjects were diagnosed with IFG, 10.97% (n = 25) with IGT and 2.19% (n = 5) with T2DM (**Table 1**).All children with T2DM were newly diagnosed upon admission.

**Table 1:** Subjects characteristic by glucose tolerance status.

	Allsubjects	Obesity*	IFG	IGT	T2DM	p	R
n (%)	228	191 (83.77)	7 (3.07)	25 (10.96)	5 (2.19)		
Sex – Male, n (%)	110 (48.25)	99 (51.83)	5 (71.43)	11 (44)	2 (40.00)		
Sex – Female, n (%)	118 (51.75)	92 (48.17)	2 (28.57)	14 (56.00)	3 (60.00)		
Age (years)	10.4 ± 3.6	10.4 ± 3.58	12.33 ± 2.78	10.88 ± 3.14	17.34 ± 0.53	<0.0001	0.31
Weight (kg)	62.7 ± 23.9	60.10 ± 23.15	77.96 ± 16.28	71.73 ± 25.53	94.26 ± 13.78	0.0005	0.28
Weight SD	2.98 ± 0.91	2.96 ± 0.90	2.8 ± 0.92	3.23 ± 1.00	2.71 ± 0.99	0.4505	0.11
Height (m)	1.47 ± 0.19	1.45 ± 0.19	1.63 ± 0.10	1.54 ± 0.19	1.67 ± 0.06	0.0016	0.26
Height SD	1.33 ± 1.23	1.30 ± 1.18	1.77 ± 1.22	1.67 ± 1.48	-0.07 ±1.29	0.0238	0.20
BMI (kg/m^2^)	27.83 ± 4.70	27.38 ± 4.34	29.92 ± 8.94	29.43 ± 4.74	33.84 ± 4.77	0.0023	0.25
BMI SD	2.98 ± 0.74	2.98 ± 0.74	2.65 ± 1.01	3.08 ± 0.72	2.82 ± 0.62	0.5621	0.10
Fatty liver on ultrasound. n (%)	51 (23.61)	36 (19.57)	3 (42.86)	9 (40.91)	3 (100)		
ALT (U/l)	25.9 ± 19.1	24.00 ± 15.41	22.83 ± 16.23	27.88 ± 12.79	85.80 ± 48.27	<0.0001	0.51
AST (U/l)	26.3 ± 10.8	25.50 ± 9.19	20.50 ± 7.97	26.33 ± 8.41	58.00 ± 21.97	<0.0001	0.48
Cholesterol (mg/dl)	166.8 ± 36.4	164.26 ± 34.48	164.29 ± 49.63	179.88 ± 37.67	207.00 ± 57.24	0.0177	0.21
HDL-C (mg/dl)	47.0 ± 14.5	47.25 ± 14.76	45.86 ± 7.95	47.91 ± 14.93	36.80 ± 5.22	0.4490	0.11
LDL-C (mg/dl)	101.5 ± 28.3	100.0 ± 27.27	98.14 ± 40.81	112.09 ± 31.10	111.40 ± 32.23	0.2195	0.14
TG (mg/dl)	105.9 ± 69.5	97.80 ± 50.34	142.57 ± 152.28	122.15 ± 54.71	284.00 ± 226.03	<0.0001	0.42
Glucose 0’ OGTT (mg/dl)	86.0 ± 8.2	84.46 ± 7.25	104.39 ± 4.03	90.36 ± 7.00	97.14 ± 2.62	<0.0001	0.51
Insulin 0’ OGTT (μIu/ml)	15.3 ± 11.9	13.74 ± 8.78	43.77 ± 39.56	17.43 ± 6.27	26.90 ± 2.02	<0.0001	0.47
Glucose 120’ OGTT (mg/dl)	114.7 ± 27.2	106.13 ± 15.99	120.90 ± 11.59	150.17 ± 11.37	224.90 ± 20.11	<0.0001	0.82
Insulin 120’ OGTT (μIu/ml)	91.4 ± 89.1	69.96 ± 52.61	121.21 ± 81.82	198.14 ± 148.11	262.56 ± 161.03	<0.0001	0.56
HOMA-IR	3.36 ± 3.01	2.90 ± 1.94	11.53 ± 10.95	3.91 ± 1.56	6.44 ± 0.34	<0.0001	0.52
Quicki	0.33 ± 0.03	0.34 ± 0.03	0.29 ± 0.04	0.32 ± 0.02	0.29 ± 0.00	<0.0001	0.37
Matsuda	5.74 ± 4.06	6.43 ± 4.12	2.52 ± 1.68	2.76 ± 1.19	1.40 ± 0.35	<0.0001	0.37

* Obesity, children with obesity with no carbohydrates metabolism disorders; BMI, body mass index; Glucose 0’ OGTT, fasting glucose; Insulin 0’ OGTT, fasting insulin; Glucose 120’ OGTT, glucose after 2 hours of oral glucose tolerance test; Insulin 120’ OGTT, insulin after 2 hours of oral glucose tolerance test.

Because of extreme values of metabolic parameters in TDM2 children (as seen in **Table 1**), they were excluded as outliers from further statistical analysis. Based on ultrasound imaging, hepatic steatosis was diagnosed in 48 patients (22.54%), of which there were 25 (22.52%) girls and 23 (22.55%) boys. The average age of subjects with steatosis was 10.71 ± 3.63 years. In the study group 11.58% (n=22) of the subjects had incorrect ALT values, whereas in the group with hepatic steatosis in USG examination ALT levels were elevated in 27.27% (n=12) of the subjects. In the whole study group the percentage of boys with elevated ALT was 14.89% (n=14), the percentage of girls was 8.33% (n=8). In 54.55% (n=12) of children with elevated alanine aminotransferase levels hepatic steatosis was found in ultrasound.

In the group of children without glucose intolerance (GI), the sonographic features of fatty liver were found in 19.57% (n = 36) of the subjects. In the group with IGT, this percentage increased to 40.91% (n = 9) subjects and in the group with IFG amounted to 42.86% (n = 3) children (**Table 1**). ALT levels were above the norm in 10.37% (n=17) of the subjects without carbohydrate metabolism disorders, 15.00% (n=3) of the IGT group and 33.33% (n=2) of the IFG group. When analyzing the occurrence of metabolic complications among the examined children, according to the pubertal development, the presence of sonographic features of fatty liver was found in 18.85% (n=23) of the prepubertal subjects. During the puberty, this percentage increased to 25.00% (n=17), and in postpubertal subjects it was 34.78% (n=8). Considering the values of liver enzymes in the prepubertal subjects abnormal ALT was noted in 11.76% (n=12) of children. During the puberty, this value ​​increased to 8.06% (n=5), while in postpubertal subjects it was 19.23% (n=5). Taking into consideration the carbohydrate metabolism disturbances among children in the prepubertal stage 1.64% (n=2) had abnormal fasting glucose and 8.20% (n=10) had impaired glucose tolerance. In the group of pubertal children, these disorders were present in 6.76% (n=5) and 14.86% (n=11), respectively. Type 2 diabetes was found in none of these groups. All subjects with DM2 were diagnosed in postpubertal children. Moreover, in the post puberty group, glucose tolerance disorders were found in 12.5% ​​of the examined children.

To asses potential cardiovascular risk, we analyzed blood pressure. Differences in systolic and diastolic blood pressure in patients depending on the concentration of alanine aminotransferase and the presence of fatty liver features on ultrasound did not show statistical significance.

Depending on the presence of hepatic steatosis there were significant differences in the values of alanine aminotransferase (p=0.0006; R 0.25), glycaemia after 2 hours of OGTT (p=0.0059; R 0.20), fasting insulin (p= 0.0006; R 0.23) and all insulin resistance indices (**Table 2**).

**Table 2:** Subjects characteristic by presence of fatty liver on ultrasound and alanine aminotransferase levels.

	Fatty liver on ultrasoundabsent	Fatty liver on ultrasoundpresent	p	R	ALT < 42U/l	ALT > 42 U/l	p	R
n (%)	165 (77.46)	48 (22.54)			168 (88.42)	22 (11.58)		
Sex – Male, n (%)	86 (77.48)	23 (22.55)			80 (85.11)	14 (14.89)		
Sex – Female, n (%)	79 (77.45)	25 (22.52)			88 (91.67)	8 (8.33)		
Age (years)	9.85 ± 3.46	10.71 ± 3.63	0.1355	0.10	10.21 ± 3.53	10.42 ± 4.15	0.7960	0.02
Weight (kg)	58.14 ± 22.56	69.20 ± 23.57	0.0034	0.20	61.54 ± 23.47	68.85 ± 26.77	0.1784	0.10
Weight SD	2.90 ± 0.89	3.28 ± 0.98	0.0111	0.17	2.97 ± 0.88	3.45 ± 1.14	0.0205	0.17
Height (m)	1.44 ± 0.19	1.50 ± 0.19	0.0538	0.13	1.46 ± 0.19	1.48 ± 0.18	0.6865	0.03
Height SD	1.28 ± 1.21	1.58 ± 1.15	0.1257	0.11	1.32 ± 1.13	1.75 ± 1.41	0.0977	0.12
BMI (kg/m^2^)	26.84 ± 4.32	29.86 ± 4.89	0.0001	0.27	27.58 ± 4.47	30.39 ± 6.14	0.0089	0.19
BMI SD	2.91 ± 0.73	3.23 ± 0.81	0.0094	0.18	2.97 ± 0.74	3.42 ± 0.81	0.0081	0.19
Fatty liver on ultrasound, n (%)	–	–	–	–	32 (72.73)	12 (27.27)		
ALT (U/l)	22.41 ± 13.64	31.37 ± 18.06	0.0006	0.25	19.91 ± 7.35	58.43 ± 16.21	<0.0001	0.82
AST (U/l)	25.19 ± 8.91	26.98 ± 9.68	0.2593	0.08	23.73 ± 7.21	38.72 ± 11.65	<0.0001	0.52
Cholesterol (mg/dl)	163.5 ± 34.6	177.6 ± 36.6	0.0196	0.16	163.5 ± 33.0	179.9 ± 41.7	0.0351	0.15
HDL-C (mg/dl)	48.21 ± 15.21	44.21 ± 10.95	0.1080	0.11	47.47 ± 14.99	43.38 ± 10.92	0.2182	0.09
LDL-C (mg/dl)	98.69 ± 26.46	111.68 ± 32.98	0.0074	0.19	99.43 ± 26.59	114.85 ± 35.29	0.0154	0.18
TG (mg/dl)	95.02 ± 49.16	123.56 ± 74.55	0.0028	0.21	98.59 ± 52.81	130.19 ± 90.68	0.0182	0.17
Glucose 0’ OGTT (mg/dl)	85.02 ± 8.08	87.79 ± 8.08	0.0384	0.14	85.10 ± 8.21	89.28 ± 7.78	0.0249	0.16
Insulin 0’ OGTT (μIu/ml)	13.12 ± 8.33	19.53 ± 17.93	0.0006	0.23	14.46 ± 10.34	22.29 ± 22.26	0.0054	0.20
Glucose 120’ OGTT (mg/dl)	108.95 ± 19.46	118.59 ± 23.72	0.0059	0.20	110.89 ± 19.93	118.43 ± 22.05	0.1099	0.12
Insulin 120’ OGTT (μIu/ml)	75.25 ± 76.28	101.88 ± 78.72	0.0411	0.15	82.39 ± 81.61	113.92 ± 79.19	0.0971	0.13
HOMA-IR	2.80 ± 1.92	4.44 ± 4.95	0.0007	0.23	3.11 ± 2.43	5.24 ± 6.32	0.0030	0.21
Quicki	0.34 ± 0.03	0.32 ± 0.03	0.0002	0.25	0.33 ± 0.03	0.31 ± 0.03	0.0041	0.21
Matsuda	6.52 ± 4.25	4.44 ± 2.92	0.0022	0.22	6.04 ± 4.18	3.92 ± 2.36	0.0246	0.17

BMI – body mass index. Glucose 0’ OGTT – fasting glucose. Insulin 0’ OGTT – fasting insulin. Glucose 120’ OGTT - glucose after 2 hours of oral glucose tolerance test. Insulin 120’ OGTT - insulin after 2 hours of oral glucose tolerance test.

**Figure 1:**
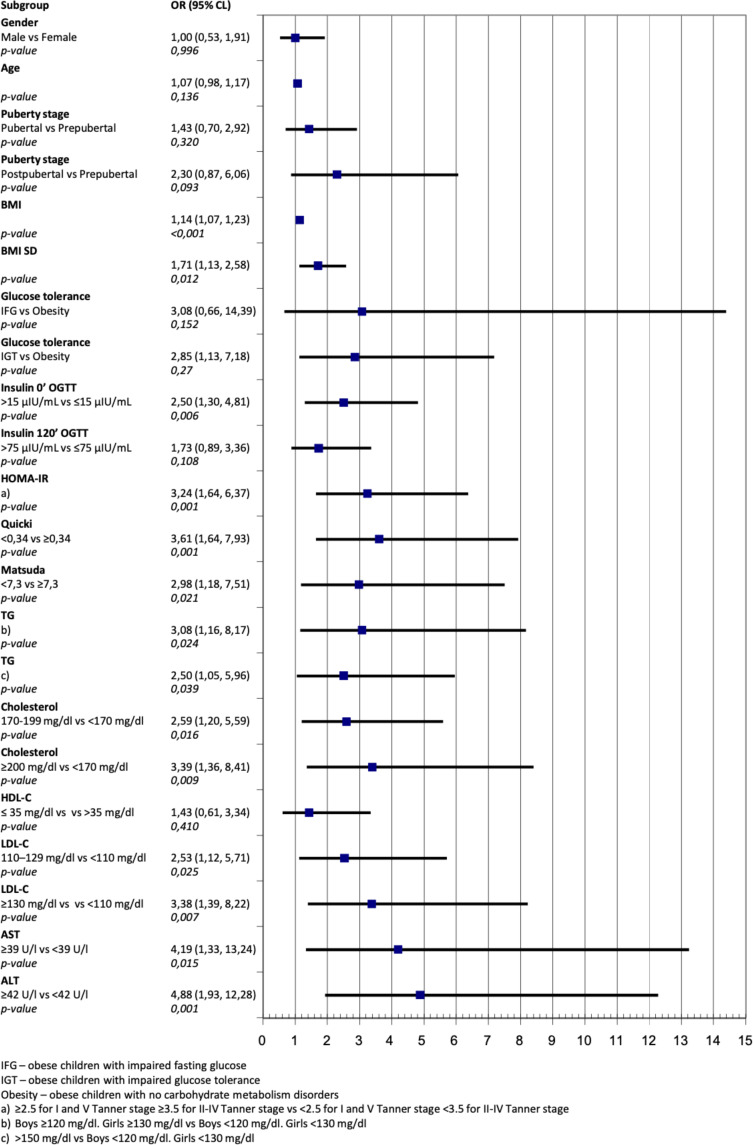
Odds ratio for fatty liver on ultrasound for different anthropometrics and biochemical variables.

The risk of abnormal liver ultrasound imaging expressed as OR in the IGT group was 2.85 times higher (95% Cl 1.13-7.18, p=0.027) compared to children with obesity without carbohydrate metabolism disorders (**Figure 1**). In case of insulin resistance expressed as HOMA-IR risk of fatty liver increased 3.24 times (95% Cl 1.64-6.37, p=0.001), 3.61 times (95% Cl 1.64-7.93, p=0.001) for Quicki and 2.98 for Matsuda (95% Cl 1.18-7.51, p=0.021)

(**Figure 1**). In subjects with high ALT values this risk was 4.88 times higher (95% Cl 1.93-12.28, p=0.001) than in subjects with normal ALT values (**Figure 1**).

**Figure 2:**
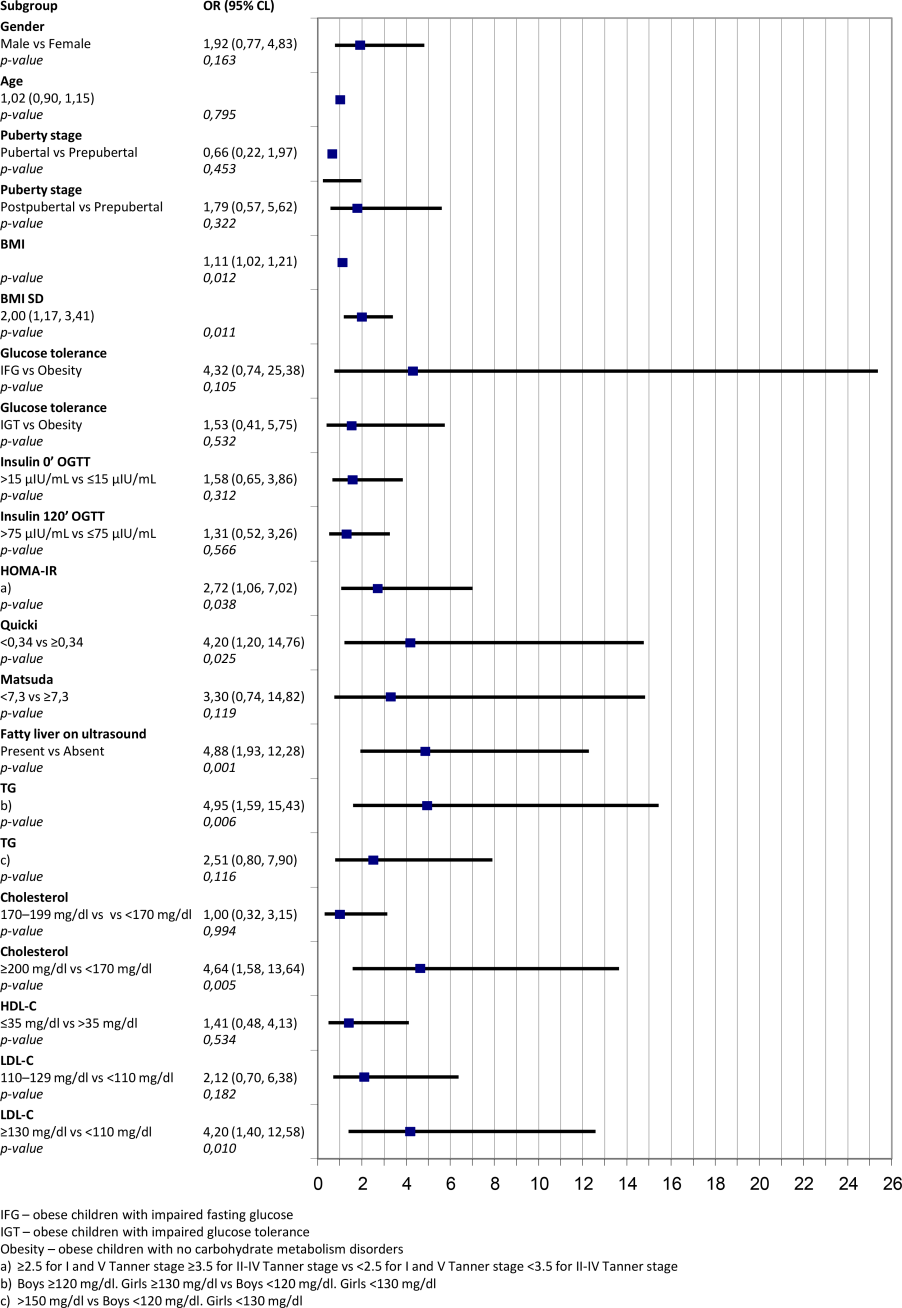
Odds ratio for abnormally high levels of alanine aminotransferase for different anthropometrics and biochemical variables.

Logistic regression for ALT shows high odds ratio for children with fatty liver on ultrasound 4.88 (95% Cl 1.93-2.28; p=0.001), for Quicki 4.20 (95% Cl 1.20-14.76; p=0.025) and HOMA-IR 2.72 (95% Cl 1.06-7.02; p=0.038) (**Figure 2**).

ALT values significantly correlated with BMI SD (r 0.34; p<0.001), insulin after 2 hours of OGTT (r 0.25; p<0.001), insulin resistance indices Quicki (r -0.24; p<0.001), HOMA-IR (r 0.24; p<0,001), Matsuda (r -0.26; p<0.001) and with cholesterol (r 0.27; p<0.001), LDL-C (r 0.23; p<0.001) and triglycerides (r 0.23; p<0.001).

Analysis of the children’s eating habits performed on part of the subjects (n=68) shows, that children’s rations were not properly balanced. Average percentage of implementation of the norm for energy and main nutrients as well as their percentage share in the diet’s energy value was exceeded in almost all analyzed nutrients except for digestible carbohydrates. Energy value of the diets was significantly varied. Average calorie intake as a percentage of suggested norm was about 30% higher than the caloric demand in 75% of the respondents. Energy intake from proteins on the suggested level of 12.5% was implemented only in less than 25% of children. Energy from fat made up for 32.2 ± 6.3% of the caloric intake (with the recommendations being 30%). Saturated fat intake exceeded the recommended norm on average by 144.1 ± 52.6%, with only 25% of analyzed children being close to the recommended goal.

## Discussion

According to data provided by CDC (Center for Disease Control and Prevention) in the last 30 years the number of children with obesity has increased twofold, while the number of obese teenagers has tripled. WHO (World Health Organization) estimations say, that in 2019 there were 38 million overweight children below the age of 5 in the world, almost half of which lived in Asia.

In the Polish population, similar to other countries in Central-Eastern Europe, which were subjected to economic and social changes, there is a visible increase in prevalence of obesity in the pediatric population. In 2007-2009 a study was performed on a random, representative population sample in the age of 7-18. The study was performed as a part of the OLAF project. According to norms set by this study, overweight and obesity was observed in 18.7% boys and 14.1% girls. In Western Pomerania a screening study concerning the prevalence of overweight and obesity was performed among children in the age 8-9 (own data, soon to be published). 4903 children have been screened, of which 22.8% had overweight and obesity – 16.5% were overweight and 6.3% were obese.

Obesity in children is a cause of many metabolic complications, including carbohydrate metabolism disorders, lipid disorders, hypertension and NAFLD. Some authors discuss a possibility of association of certain obesity phenotypes with a different cardiovascular risk (20). It may be argued, that the same phenotypes may be associated with an increased risk of NAFLD development.

Early data publish in NEJM in 2002 suggest the prevalence of impaired glucose tolerance in 25% of children with obesity in the age of 4-10 and 21% obese teenagers in the age of 11-18. In this study “silent type 2 diabetes” was diagnosed in 4% of obese teenagers (21). Italian studies on increasing incidence of carbohydrate metabolism disorders in children with obesity show that those disorders were found in 12.4% of patients. The most common disorder was impaired glucose tolerance, found in 11.2% of patients. It was more common in teenagers than in children (14.8% vs. 4.1%). “Silent T2DM” was diagnosed in two teenagers (0.4%) (22).

In presented analysis impaired fasting glucose was present in 3.07% (n=7) of the subjects, impaired glucose tolerance in 10.97% (n=25); in 2.19% (n=5) of subjects based on OGTT type 2 DM was diagnosed, which may be considered “silent T2DM”. Despite varying data concerning different populations, increasing prevalence of carbohydrate metabolism disorders in children with obesity remains a fact. To quantify the grade of insulin resistance, HOMA-IR, Quicki and Matsuda indices were used in this study, as well as fasting insulin levels. As expected, HOMA-IR was high in children with type 2 DM. Surprisingly high values of HOMA-IR were also found in children with impaired fasting glucose (**Table 1**). What certainly draws attention while analyzing biochemical studies’ results of patients with carbohydrate metabolism disorders are elevated aminotransferase levels, especially ALT, which was highest in children with T2DM (**Table 1**). Because other biochemical parameters in T2DM children were also significantly different from the rest of the analyzed group, they were considered outliers and excluded from further statistical analysis.

Another important complication of obesity is nonalcoholic fatty liver disease. It is defined as accumulation of fat in the liver in the absence of excessive alcohol consumption or other liver pathologies (23). Spectrum of the disease ranges from steatosis to steatohepatitis, which is characterized by hepatocellular inflammation and injury, progressing to fibrosis and eventually cirrhosis (24, 25). NAFLD is one of the most common causes of chronic liver disease in childhood (26).

The incidence of NAFLD is hard to estimate, both in the adult and pediatric population. The golden standard of diagnosing NAFLD is liver biopsy (25), which difficult to use in everyday practice – hence the search for other, less invasive methods, such us checking the serum levels of inflammation markers and assessing the liver in imaging studies such as ultrasonography or *magnetic resonance imaging* (MRI). In light of the most recent guidelines, the idea of liver biopsy being the golden standard in NAFLD diagnostics need to be re-evaluated – while it indeed remains the diagnostic gold standard for the assessment of fibrosis and inflammation, it is not recommended in simple hepatosteatosis (27). Moreover, biopsies have known limitations and possible disadvantages which ring especially true in the pediatric population - a risk of complications due to the procedure being invasive, sampling variability because of the small sample obtained, and the heterogeneous distribution of histological changes in liver parenchyma. As pointed out by the authors, routine liver biopsies performed specifically to confirm NAFLD seem unwarranted due to the prevalence of the condition, and may even be considered unethical. While liver biopsy remains the only diagnostic tool to confirm non-alcoholic steatohepatitis (NASH), its prevalence among NAFLD patients is quite low - estimated at 1.5%-6.5%. Considering these points, authors suggest, that liver biopsies should only be performed in patients at high risk of the progressive type of the disease (28, 29).

In existing publications, the prevalence of NAFLD was estimated to be somewhere in a wide range between 1.7% to 85% of children with obesity (30–33). Such a wide range depends on the analyzed population and chosen diagnostic methods. In an analysis by American authors considering 408 children with obesity in the mean age of 13.2, NAFLD was found in 26% of children, more often in boys 29.4% than girls 22.6% (34). Meta-analysis presented by Anderson et al. estimates the incidence of NAFLD in the general population at 7.6%, and 34.2% in children with obesity (35).

In available literature several studies can be found, where biopsy was used as a diagnostic criterium. Schwimmer et al. published a report in which based on autopsy studies of 742 children in the age of 2-19 hepatic steatosis was found in 9.6% of children with normal weight and 38% of children with obesity (36). One of the imaging studies used to assess the grade of hepatic steatosis is magnetic resonance (MRI), but because of the costs of the study ultrasonography is used more often (35, 37). Hernaez et al. in their analysis have found ultrasonography to be accurate and reliable for detection of moderate-sever fatty liver (sensitivity 84.8%, specificity 93.6% compared to histology) (38). As opposed to adult recommendations by EASL-EASD-EASO (10), NASPHGAN does not recommend ultrasound as a screening for NAFLD in children. It opens the area for further discussion, as ultrasound, being a cheap, simple, non-invasive and repetitive examination, which would make it a suitable choice for the pediatric population. It is also undeniable, that the constant technological progress in the radiology department improves the result achieved by the examinations.

In our study hepatic steatosis was found in 22.54% of the subjects. This prevalence is lower than previously reported by other authors. It may be caused by the young age of the whole study group and the fact, that 122 of the children were prepubertal. Even though they were already overweight, they may have not yet presented with NAFLD as a complication of obesity.

As reported by Younossi, glucose intolerance, and especially T2DM, is an accelerator of NAFLD and a predictor of severe fibrosis and mortality (39). In our study we tried to assess the fraction of children with NAFLD according to the grade of glucose metabolism disorders. In children without carbohydrate metabolism disorders features of fatty liver were found in 19.57% of the subjects, while in children with glucose intolerance 40.91% of the subjects were affected. All of the children with type 2 diabetes were diagnosed with hepatic steatosis. Increasing percentage of children with hepatic steatosis in groups with glucose intolerance undoubtedly shows a correlation between carbohydrate metabolism disorders and fatty lived disease. Odds ratio (OR) calculations show, that the risk of hepatic steatosis is, 2.85 times higher in children with impaired glucose tolerance.

In our study there is no noticeable difference in occurrence of hepatic steatosis in ultrasound examination when it comes to children’s sex (22.55% vs 22.52%), which is consistent with the study by Prokopowiczet al. (40)

In pediatric populations, a meta-analytic study showed that the pooled NAFLD prevalence is higher in boys than in girls in general populations and obese clinical cohorts (41). The study also revealed significant variance across the published reports, which is partly explained by the technique used to diagnose NAFLD (ultrasound versus aminotransferases) and by failure to consider pubertal stages when sex hormone levels change dramatically in a sex-specific manner (41). According to the meta-analysis by Anderson et al. (35), the incidence of hepatic steatosis based only on USG is similar in both sexes, whereas considering only ALT, it is more common in boys. Our findings in the presented study support similar results. Based on ultrasound imaging the prevalence of hepatic steatosis doesn’t differ between the sexes, while based on ALT it is more common in boys, that difference is however not statistically significant.

Many studies support the central role of insulin resistance in the development of fatty liver (42). The presence of liver steatosis is an important marker of multiorgan insulin resistance, independently of BMI, body fat percentage, and visceral fat mass (43). In particular, NAFLD has been found to be associated with insulin resistance in liver, skeletal muscles and adipose tissue in children and adolescents with obesity (44).

In our study high values of insulin resistance indices QUICKI, HOMA-IR, Matsuda significantly increase the risk of hepatic steatosis (OR 3.61; 3.24 and 2.98 respectively, p<0.001). In the presence of elevated fasting insulin levels, the risk of hepatic steatosis was also significantly higher – OR 2.50 (p=0.006).

According to NASPGHAN guidelines, serum ALT levels should be used as a screening tool for assessing the risk of NAFLD, with normal values <22 U/l for girls and <26 U/l for boys (3). In our analysis local laboratory’s norm was used, with values above 42 U/l determined as too high. In the study group 11.58% of children (n=22) had elevated ALT levels. In the group with fatty liver features in USG examination those values were elevated 27.27% of children (n=12). In the group of children with high ALT levels 54.55% of children (n=12) had features of fatty liver. It must be noted, however, that the mean value of ALT levels in the whole study group was 24.37±15.16 U/l, which exceeds the values given as reference by NASPGHAN in all analyzed subgroups. Especially high levels were observed in the group of children with impaired glucose tolerance **Table 1**). In previously cited study by Yu et al. optimal cut-off points for ALT have been established according to sex as 42 U/l for boys and 30 U/l for girls (34).

In our analysis in 10.37% of the subjects with no carbohydrate metabolism disorders ALT values were abnormal. The percentage of children with abnormal ALT levels in IGT group was 15.00%, in IFG group 33.33% and in T2DM group 80.00%. Meta-analysis published by Anderson et al. shows, that in 14 clinical studies concerning children with obesity abnormal ALT values were observed in 13.7% of children (6.2 to 27.6%) (35). It is emphasized, that using ALT to diagnose NAFLD may cause overdiagnosis of this ailment in persons with normal body weight, as well as underestimation of this problem in patients with obesity.

In the proprietary study ALT levels correlated with insulin after 2 hours of OGTT, insulin resistance indices as well as triglyceride and cholesterol levels.

The assessment of odds ratio shows, that the highest risk of abnormal ALT values concerns children with high insulin resistance indices, hypertriglyceridemia and hypercholesterolemia. OR values for elevated ALT levels and fatty liver features in sonograms is 4.88 (95%Cl 1.93-12.28, p=0.001). Considering adipose tissue is a hormonally active tissue, some adipokines may be associated with the metabolic state of the child – as such, the leptin/adiponectin ratio may be a useful indicator of insulin resistance (45).

Routine work with a child with obesity at risk of NAFLD always involves cooperation with a dietitian. In our analysis, declared energy consumption presented as a percentage of the norm were higher by about 30% in relation to the caloric need in as many as 75% of the subjects. Declared consumption of simple carbohydrates amounted to 112.4±68.9% of the norm.

An analysis of quantity and quality of products consumed by the child, as well as eating habits correction are crucial. Latest studies show, that diet rich in saccharose and HFCS not only increases the risk of NAFLD, but also NASH (46, 47). Excessive consumption of fructose and saccharose correlates with epidemic increase of obesity, metabolic syndrome and NAFLD occurrence (48). Long-term excessive fructose consumption leads to leptin resistance and, as a consequence, obesity (49). Anika Nier et al. in their study concerning the effect of fructose consumption and nutrition patterns on the development of NAFLD in children with obesity emphasize, that children with obesity and early symptoms of NAFLD have a higher energy intake in comparison to children without hepatic steatosis, mostly caused by fructose and saccharose consumption (6). The most recommended way of preventing and treating obesity is still taking measures to change the patient’s lifestyle, including modification of the eating habits and increasing physical activity.

What we consider to be the strength of the study is it being performed on a group of children, considering the scarcity of publications on the subject of NAFLD in the pediatric population. Furthermore, the group consisting of consecutive children from one region of Poland guarantees the subjects’ homogeneity (only Caucasian origin), without placing bias on the study group by selecting specific patients. The most important limitation of our study is a relatively low number of subjects in each sub-group, with type 2 diabetes. It is known though, that for type 2 diabetes to develop a longer history of obesity is needed. In our opinion, supported by other authors’ research, those patients require special attention. In the future they are possible candidates for combined pharmacological or bariatric treatment (50).

The study was also performed on a group of children in a wide range of age (2-18), which may cause the results to be skewed by the effect of sex hormones in older children. Metabolic complications of obesity, such as fatty liver, hypertransaminasemia and disturbances in glucose metabolism, are significantly more common in older children and with advanced puberty. DM2 only occurred in children with Tanner stage V.

Only ALT and AST were used as markers of liver function. However, they are considered to be the first routinely performed assessment of liver function in children, who (if required) went on to be diagnosed further. Furthermore, studies evaluating GGTP as a marker of hepatic steatosis in children have not yet been performed. One of the aims of the study was to assess if routinely performed laboratory tests, i.e. ALT and AST, could be considered sufficient to diagnose NAFLD in those children. What needs to be noted is that the study is a clinical analysis performed by pediatric endocrinologists in children diagnosed because of obesity, with no assumptions being made as for the presence of liver dysfunction beforehand, which is why the tests performed during diagnostics are not meant to specifically focus on that subject. The list of possible complications of obesity is immeasurably long, meaning that diagnosing each and every system in the patient’s body during one visit in the outpatient’s clinic is technically impossible, which forces physicians to look for the most effective screening studies possible.

One of the screening tests that may be an indicator of both NAFLD and cardiovascular risk may be waist to height ratio. In the analysis of Umano and al (51). it has been proven that WHR can be a good screening anthropometric parameter for the assessment of NAFLD. Blood pressure measurement is essential to assess cardiovascular risk in obese subjects. In our analysis differences in systolic and diastolic blood pressure in patients depending on the concentration of alanine aminotransferase and the presence of fatty liver features on ultrasound did not show statistical significance.

Further research is required with other variables considered, such as dividing the children in sub-groups according to the stage of liver steatosis. Novel diagnostic methods may also play a bigger role in NAFLD diagnostics, however further research is necessary, as not all innovative techniques will gain success in the clinical and research area. Reevaluation of ALT normative values seems necessary, which requires them to be included in later studies.

## Conclusions

Almost every fourth child with obesity in the study group presents features of fatty liver in ultrasound examination. Although ultrasound is not recommended by NASPGHAN for the diagnosis of NAFLD in children, it allows identifying a high percentage of children with features of fatty liver. This percentage increases significantly in children with glucose intolerance. Routinely used ALT reference values may be responsible for a high percentage of subjects with normal ALT levels despite the presence of hepatic steatosis in ultrasound.

## Data availability statement

The original contributions presented in the study are included in the article/supplementary material. Further inquiries can be directed to the corresponding author.

## Ethics statement

The studies involving human participants were reviewed and approved by Pomeranian Medical University Bioethical Committee on April 14 2004, decision number BN-001/67/04. Written informed consent to participate in this study was provided by the participants’ legal guardian/next of kin.

## Author contributions

Conceptualization, KM, EP and MW; methodology, KM, EP, and AH-J; software, TJ and AB-M; validation, KM, EP and MW; formal analysis, KM and KS; investigation, KM, KS and EP; resources, KM, KS and AB-M; data curation, KM and KS; writing—original draft preparation, KM, EP and AH-J; writing—review and editing, TJ, EP, AB-M and MW; visualization, TJ and AB-M; supervision, EP and MW; project administration, KM and EP. All authors contributed to the article and approved the submitted version.

## Conflict of interest

The authors declare that the research was conducted in the absence of any commercial or financial relationships that could be construed as a potential conflict of interest.

## Publisher’s note

All claims expressed in this article are solely those of the authors and do not necessarily represent those of their affiliated organizations, or those of the publisher, the editors and the reviewers. Any product that may be evaluated in this article, or claim that may be made by its manufacturer, is not guaranteed or endorsed by the publisher.
